# Can AI Write MCQs? A Comparison of ChatGPT-Generated and Educator-Authored Multiple-Choice Questions in Graduate Nursing Education

**DOI:** 10.3390/nursrep16090304

**Published:** 2026-08-27

**Authors:** Nader Alnomasy, Habib Alrashedi, Maha Dardouri, Soha Kamel Mosbah Mahmoud, Petelyne Pangket, Sang Bin You, Aluem Tark, Deborah Becker, Richard Balacuit Maestrado, Romeo Mostoles, Rayhanah R. Almutairi, Ebtsam Abouhashish, Sudharani B. Banappagoudar, Asim A. Alreshidi, Waleed Meajib Alshammari, Faisal Fahaad Alrshidi, Razan Alsayed, Sharifah Alsayed, Jiyoun Song

**Affiliations:** 1 Medical Surgical Nursing Department, College of Nursing, University of Hail, Hail 81451, Saudi Arabia; 2 Community Health Nursing Department, College of Nursing, University of Hail, Hail 81451, Saudi Arabia; 3 Department of Community and Mental Health Nursing Sciences, Taif University, Taif 21944, Saudi Arabia; 4 Department of Biobehavioral Health Sciences, University of Pennsylvania School of Nursing, Philadelphia, PA 19104, USA; 5 Department of Nursing, Helene Fuld College of Nursing, New York, NY 10035, USA; 6 Department of Psychiatric and Mental Health Nursing, College of Nursing, University of Hail, Hail 81451, Saudi Arabia; 7 Community and Psychiatric Mental Health Nursing Department, College of Nursing, Princess Nourah Bint Abdulrahman University, Riyadh 11671, Saudi Arabia; 8 College of Nursing, King Saud Bin Abdulaziz University for Health Sciences, Jeddah 21423, Saudi Arabia; 9 King Abdullah International Medical Research Center, Jeddah 22384, Saudi Arabia; 10 Ministry of National Guard Health Affairs, Jeddah 22384, Saudi Arabia; 11 Faculty of Nursing, Alexandria University, Alexandria 21526, Egypt; 12 Department of Nursing, College of Applied Medical Sciences, King Faisal University, Al Ahsa 31982, Saudi Arabia; 13 Emergency Department, King Khalid Hospital, Hail Health Cluster, Hail 55421, Saudi Arabia; 14 Advanced Oncology Nursing, King Faisal Specialist Hospital & Research Center, Riyadh 11211, Saudi Arabia; 15 Department College of Medicine, Sechenova University, Moscow 119048, Russia; 16 Nursing Education Department, King Fahd Armed Forces Hospital, Jeddah 23311, Saudi Arabia

**Keywords:** artificial intelligence, ChatGPT, large language models, multiple-choice questions, graduate nursing education

## Abstract

**Background/Objectives:** High-quality multiple-choice questions (MCQs) are essential for valid assessment in graduate-level nursing programs, yet developing such items is resource-intensive for educators. Large language models such as ChatGPT may generate educational assessment content; however, evidence regarding their effectiveness in producing graduate-level nursing examination items remains limited. This study compared the perceived quality of ChatGPT-generated and educator-authored MCQs for graduate-level nursing examinations and examined exploratory associations between assessor characteristics and quality ratings. **Methods:** A comparative cross-sectional study was conducted among 27 international nurse educators who independently evaluated 50 MCQs (25 ChatGPT-generated and 25 educator-authored) using an expert-reviewed rubric assessing eight quality domains, with the primary outcome being the between-source difference in perceived quality ratings. Participants were blinded to question source. **Results:** ChatGPT-generated MCQs received nominally higher ratings than educator-authored items across most domains, but effect sizes were uniformly small (Cohen’s *d* = 0.11–0.33). No significant differences were observed in perceived difficulty or distractor plausibility; after Bonferroni correction for multiple domain comparisons, differences in cognitive level, scenario relevance, and instructional alignment remained significant. **Conclusions:** ChatGPT-generated MCQs achieved perceived-quality ratings broadly comparable to educator-authored items when supported by structured prompting and expert refinement, although expert review remains necessary before classroom use, and these results should be regarded as preliminary. These findings support AI-assisted item development as a complementary strategy pending replication in larger, more rigorous studies.

## 1. Introduction

Valid and reliable assessment is essential for graduate nursing education. In these courses, examinations are expected to assess a student’s ability to use their nursing knowledge in clinical scenarios and make complex decisions about the care of their patients [1]. Graduate nursing courses were chosen as the subject of this study as they typically assess nursing students for higher-order clinical reasoning skills rather than the knowledge of the profession that is learned in undergraduate nursing courses. The definition of graduate nursing education in this study is the coursework within Master of Science in nursing programs or other advanced practice programs. These programs commonly rely on multiple-choice questions (MCQs) to evaluate students’ knowledge and clinical skills. Doctoral-level programs, by contrast, tend to place more weight on scholarly writing than on MCQ-based testing, though this distinction is not absolute; it is offered here simply as a rationale for the study’s scope rather than as an established empirical claim. Therefore, graduate nursing programs are the most appropriate focus for this study, as the comparison between AI- and educator-created MCQs will be most relevant to those courses.

MCQs are one of the most commonly utilized assessment methods in health professions education programs today. The efficiency with which they can be used to assess a large number of students in the subject matter makes them ideal for assessing large cohorts of students [2]. Yet writing a good MCQ in the first place is another matter: producing high-quality items takes considerable faculty time, subject-matter expertise, and several rounds of review. Additionally, MCQ examinations are also time-efficient for the development of question items that can assess multiple learning objectives and skills. However, the creation of high-quality MCQs that move beyond the assessment of knowledge to the assessment of more advanced cognitive skills is a challenging task that requires the expertise and skills of the question creators [3]. The increasing number of students enrolled in graduate nursing programs and the increasing amount of coursework that faculties must develop for their classes pose an additional challenge to the creation of quality assessment items.

While MCQs may be widely used in the health professions, the quality of MCQs and the ability of the examination to assess the knowledge and skills of students are frequently the subject of criticism when these examinations are created with poor attention to detail. Common errors in the creation of MCQ examinations are common and often lead to assessments that are not valid or reliable for their students [2,4]. Quality MCQs require that attention be paid to a number of considerations in their development, including the cognitive level that the question should be assessing, the distractors for the questions, the language of the question, and whether the question is aligned with the learning outcomes for the course in which the examination will be administered [3]. The creation of such quality MCQs can be time-consuming for educators, which has made it possible for AI technologies to be implemented into the process of developing MCQs for health professions education programs.

Within graduate-level health professions education programs, it is expected that examinations will assess the higher-order cognitive processes of students rather than the knowledge that students possess of the course material [3,5]. In the field of nursing education in particular, such knowledge is referred to as clinical reasoning, which requires that students integrate their nursing knowledge and skills in the care of their patients [6,7]. Well-written MCQs are capable of assessing these clinical reasoning skills by presenting realistic scenarios to the students in which they are required to make the decisions about the care of their patients [8]. The quality of these MCQs will depend upon various factors, including the cognitive level that the questions should be assessing, the realistic nature of the clinical scenarios, the distractors that are used for each question, the language that is used in the questions, and the extent to which these items are aligned with the learning outcomes for the course [1].

Large language models (LLMs), such as ChatGPT, have the potential to support the tasks that are required for health professions education programs, such as the creation of examination questions [9,10]. The use of AI in nursing education has grown rapidly in recent years, and a review of the literature on this subject reveals a number of innovative uses of this technology within nursing education programs [9]. The use of LLMs to support health professions education programs has been found to reduce the time that faculty spend creating examination questions for their classes [10]. However, it has also been found that many of the questions created by these AI technologies contain inaccuracies in their content, include implausible distractors for the examination items, and are not as sophisticated in assessing the cognitive skills of the students as those created by experienced educators [11]. Therefore, it is required that a comparison between these two types of examinations be performed in order to determine the relative merits of each.

The development of examination items in health professions education programs requires the use of experts in the field of nursing, peer review of the developed questions, and psychometric evaluation of the completed examinations [2,4]. Many studies have been performed in the literature regarding the quality of examination items in health professions education programs. However, few of these studies have examined the influence of the educators who are reviewing these examination items on their assessments of the quality of each item [3,4]. The understanding of these factors is essential in the analysis of these examination scores and in the development of effective educational programs for health professions faculty members who will be utilizing these technologies [9].

Many studies have examined whether the use of AI in the development of examination items in the health sciences has limitations in the higher-order cognitive skills that can be assessed by those examination items [11,12,13,14]. Some studies have found that the examination items created by AI technologies were able to be correlated with those created by human experts in the field [15,16,17]. However, other studies have found that these examination items have limitations in assessing some cognitive skills and in their assessment of learning outcomes for the health sciences program in which those examinations will be administered. Therefore, further study is required to determine whether the examination items created by AI technologies are able to effectively assess the graduate-level skills of nursing students as compared to those created by experienced educators in the field of nursing.

The present study aims to perform a comparison between examination items created by large language models such as ChatGPT and those created by experienced educators in graduate nursing programs [1]. The present study will allow researchers to evaluate the quality of each examination item within the following parameters: cognitive complexity and the ability of the examination to assess the higher-order skills of the students [3,5,6,7,8], the relevance of the scenario to the students’ nursing practice, the appropriateness of the instructional objectives that the examination should be assessing, the language in which the questions are written and the inclusivity of their content, and the effectiveness of each type of examination in assessing the learning of the graduate-level nursing students.

Within the literature on the development of examinations for health professions education programs, a number of topics have been explored in depth, including the quality of the examination items and the outcomes of the examinations [2,4]. Furthermore, various studies have explored the skills of the students in their use of those examinations to assess their knowledge of the field of nursing [1]. However, there are at least two areas in which the literature on the development and assessment of examination items has failed to explore topics of significant interest to the current field. One of these areas is that while there is an expanding amount of research on the comparison of AI-authored examination items with those written by educators, little research has been performed in the area of graduate nursing education [9].

Additionally, another area in which research has not explored topics of interest in the creation of examinations for these fields is the role of the evaluators in the examination process [18,19]. Few studies have explored how the characteristics of the educators who review the examinations, including the age and experience of the educators, affect the review of those examination items. Most of the studies that have been performed on this subject have explored the ability of the AI technologies to perform as well as the educators in the field of nursing and not how the educators respond to those examination items and the technology that is utilized to create them. Additionally, research on the impact of the characteristics of educators on the assessment of AI-authored examination items has not yet been explored in the literature. By addressing these research areas, valuable information will be collected that can be used to improve the assessment practices used in the field of graduate nursing education.

The primary objective of this study was to compare the perceived quality of ChatGPT-generated and educator-authored MCQs for graduate nursing examinations across eight quality domains, including scenario relevance, cognitive complexity, and instructional alignment. A secondary, exploratory objective examined whether assessor characteristics—age and teaching experience—were associated with quality ratings; given how few participants fell into some of these subgroups, this part of the analysis is treated as hypothesis-generating rather than confirmatory.

Most prior studies evaluating AI-generated MCQs have focused on undergraduate or medical education. The present study instead centers on graduate nursing education and combines a blinded expert evaluation design, a study-developed multidimensional assessment rubric, and a test of educators’ ability to distinguish AI-generated from educator-authored questions. Together, these elements provide a more comprehensive evaluation of AI-assisted assessment development in this population than has been reported previously.

Graduate nursing assessments differ from undergraduate examinations in placing greater emphasis on clinical reasoning, evidence-based decision-making, leadership, ethical judgment, and complex patient management. These higher-order competencies were considered a more demanding test of AI-generated assessment quality than factual recall alone, which is why this study centers on graduate-level rather than undergraduate nursing education.

## 2. Methods

### 2.1. Study Design

A comparative cross-sectional design was used in this study to evaluate the quality of ChatGPT (GPT-4)-generated and educator-authored MCQs for graduate-level nursing examinations. The primary outcomes were the between-source differences in perceived MCQ quality ratings across eight quality domains. Secondary exploratory outcomes were the associations of assessor age and teaching experience with quality ratings. What about source-identification accuracy?

### 2.2. Participants and Sampling

Nurse educators were recruited using purposive sampling through the authors’ professional networks, nursing education institutions, and relevant mailing lists. Eligible participants were required to hold at least a graduate degree in nursing, currently teach or have recently taught in a graduate nursing program, and have experience developing nursing course content or assessments. No minimum age, geographic location, specialty, or length of teaching experience was required for eligibility. Recruitment occurred between 15 July 2025 and 20 February 2026.

A total of 27 nurse educators participated in the study. Participants represented diverse geographic regions, including the Middle East, North America, Asia, Europe, Africa, and Australia, as well as a range of nursing specialties. Nine participants specialized in medical-surgical nursing and four in nursing administration, leadership, and management. Other represented specialties included adult health and advanced practice nursing, nursing informatics, maternal–neonatal nursing, critical and burn care nursing, and infectious-disease nursing.

Because invitations were disseminated through multiple professional networks, institutions, and mailing lists without a fixed sampling frame, the total number of individuals who received the invitation could not be determined. Therefore, an overall response rate could not be calculated. This limitation is addressed in Section 4.6.

The MCQs evaluated in this study were developed by members of the research team and external nursing instructors. To minimize potential conflicts of interest and assessment bias, neither the study authors nor the MCQ item writers participated as evaluators of the questions. One participant’s highest qualification was a Bachelor of Science in Nursing (BSN), although the prespecified eligibility criteria required at least a graduate degree in nursing. This participant met the remaining eligibility criteria, including current or recent graduate-level teaching and experience in developing course content and assessments, and was retained in the study. This protocol deviation is acknowledged in Section 4.6 as a limitation.

Due to the use of expert panelists rather than the broader nurse educator population, no formal a priori power analysis was conducted. The study was designed to obtain expert evaluations of AI- and educator-generated assessment items rather than to estimate population-level effects. Accordingly, a target sample of 25–30 graduate nurse educators with relevant item-writing and assessment experience was established based on comparable blinded expert-panel evaluations of AI- and human-generated assessment items in the health-professions literature (CITE). Given the resulting sample size, subgroup analyses by age and teaching experience are treated as exploratory given the resulting small cell sizes.

The final sample reflected eligible nurse educators from international nursing programs who had relevant experience in graduate nursing education and assessment development and voluntarily agreed to participate. Analyses examining associations between educator characteristics and MCQ ratings were therefore considered exploratory and hypothesis-generating.

To maintain independence in the evaluation process, individuals involved in writing the educator-generated items did not participate in evaluating the study items, and evaluators were blinded to whether each item was AI- or educator-generated. The research team responsible for generating or compiling the AI-generated items did not participate in item scoring.

### 2.3. Materials: MCQ Set

The assessment set comprised 50 MCQs that were divided equally into two groups: 25 ChatGPT-generated MCQs and 25 educator-authored MCQs. The ChatGPT-generated MCQs were created based on graduate nursing course objectives and targeted cognitive levels. The questions were revised based on prompts sent to ChatGPT. The educator-authored MCQs were created by nurse educators who shared the same graduate nursing course objectives as the prompts that were sent to ChatGPT for the generation of the AI-generated MCQs. Each educator-authored MCQ was reviewed for its relevance and appropriateness with its respective prompt before being included in the assessment. The prompts sent to the human item writers are provided in Supplementary Section S1 and the prompt templates for the AI-generated MCQs are provided in Supplementary Section S3.

### 2.4. Quality-Assessment Rubric

The assessment set consisted of 50 MCQs that were divided equally into two groups: 25 ChatGPT-generated MCQs and 25 educator-authored MCQs. The ChatGPT-generated MCQs were created based on graduate nursing course objectives and target cognitive levels. The questions were revised based on prompts sent to ChatGPT. The educator-authored MCQs were created by nurse educators who shared the same graduate nursing course objectives as the prompts that were sent to ChatGPT for the generation of the AI-generated MCQs. Each educator-authored MCQ was reviewed for its relevance and appropriateness with its respective prompt before being included in the assessment. The prompts sent to the human item writers are provided in Supplementary Section S1 and the prompt templates for the AI-generated MCQs are provided in Supplementary Section S3.

No rubric existed that could accurately evaluate the domains for the MCQs created for this study. As a result, a rubric was developed for this study. Existing rubrics for the quality of MCQs were incorporated into the rubric that was developed for this study [2,4,20,21]. The rubric for this study contained eight domains that were evaluated using a Likert scale of 1–5 (1 = very poor, 5 = excellent). The eight domains were cognitive level, scenario relevance, perceived discrimination potential, perceived difficulty, distractor plausibility, bias and inclusive language, clarity and precision, and instructional alignment. Prior to data collection, the rubric underwent expert review and within-study reliability testing. These procedures support the reliability of the instrument; however, external validation has not yet been established. Domain definitions and 1–5 rating anchors are summarized in Section S2 (Supplementary Materials). Before evaluation, all MCQs from both sources underwent a common formatting-standardization pass covering layout, numbering, and single-best-answer verification (Section 2.5). Beyond this shared step, the two sources were not developed through an identical review process: ChatGPT-generated items underwent iterative prompting and expert refinement (Supplementary Section S3), whereas each educator-authored item was reviewed once by its writer for relevance and appropriateness to the corresponding prompt. This difference in the extent of refinement may have contributed to the observed difference between AI- and educator-generated items and is acknowledged as a limitation in Section 4.6.

### 2.5. Blinding Procedure

All MCQs were reformatted into a standardized template with an identical layout, numbering, font style, answer structure, and presentation format. A single randomized sequence of the 50 MCQs was generated using Qualtrics (2025–2026 version, Qualtrics, LLC, Provo, UT, 84604, USA) before data collection and presented identically to all participants. Participants were informed only that they would evaluate graduate-level nursing MCQs using a standardized rubric. This was intended to minimize assessor bias associated with expectations regarding either AI-generated or educator-authored content.

### 2.6. Source Identification Assessment

Following completion of the quality ratings, participants were asked to indicate, for each MCQ, whether they believed the item had been generated by ChatGPT or authored by a human educator. The source-identification task was completed after and separate from the rubric ratings so that it would not influence the quality evaluations. Responses were coded as binary classifications indicating whether the individual identified the question as having been created by ChatGPT or an educator. Participants were presented with a classification item after each MCQ to make this choice. Each classification was compared to the true origin of the question to determine the percentage of correct classifications by the participants. This measure of the participants’ accuracy in identifying which type of author created the question provided an understanding of whether educators were capable of distinguishing between human- and AI-created questions when both were presented blindly to them.

### 2.7. Statistical Analysis

For the Likert-scale ratings, each rating was treated as numeric. Between-source comparisons between ChatGPT and educator-created MCQs were performed using independent-samples *t*-tests. Additionally, comparisons between the different age and experience groups were made using one-way analysis of variance (ANOVA). The relationships between the suitability of the MCQs and their source were determined using chi-square tests. Missing values (<3.5% of the 1350 total responses) were imputed by taking the mean of the items within each MCQ. Along with statistical significance tests, effect sizes were calculated to determine the size of the statistical relationship between the different variables. Cohen’s *d* was used to determine the effect sizes of the between-source comparisons, while eta squared was calculated for the analyses involving the assessor characteristics. As the ratings within each MCQ were made by different assessors, and within each MCQ, the MCQs themselves were different, it is important to consider that the observations were not independent of each other: the 1350 ratings reflect 27 assessors each rating the same 50 MCQs, so ratings are nested within both assessors and items. Thus, the results of the *t*-tests and ANOVAs performed should be interpreted as exploratory in nature rather than confirmatory.

Because the present study was designed as an exploratory expert-panel evaluation, mixed-effects modelling was not prespecified and therefore fell outside the planned statistical analysis. Given that repeated ratings were nested within assessors and MCQs, the reported inferential analyses should be interpreted as exploratory rather than confirmatory. The absence of mixed-effects modelling is acknowledged as a limitation in Section 4.6.

To address multiple comparisons and improve the precision of the reported estimates, 95% confidence intervals were calculated for each Cohen’s *d* using the standard large-sample approximation. A Bonferroni-adjusted significance threshold of α = 0.00625 (0.05/8) was applied to the eight domain comparisons reported. For other exploratory analyses, statistical significance was evaluated at *p* < 0.05 (two-tailed), with findings interpreted cautiously in view of the study design and sample size.

Overall source-identification accuracy was formally compared with the chance level of 50% using a one-sample proportion test. Because this pooled analysis does not account for the nesting of ratings within assessors and MCQs, the result was interpreted as exploratory and was not considered a substitute for a mixed-effects analysis.

Missing data represented less than 3.5% of the 1350 ratings and were addressed using item-level mean imputation. A complete-case sensitivity analysis was not performed. The potential influence of the imputation approach is therefore acknowledged as a limitation in Section 4.6.

**Figure 1:**
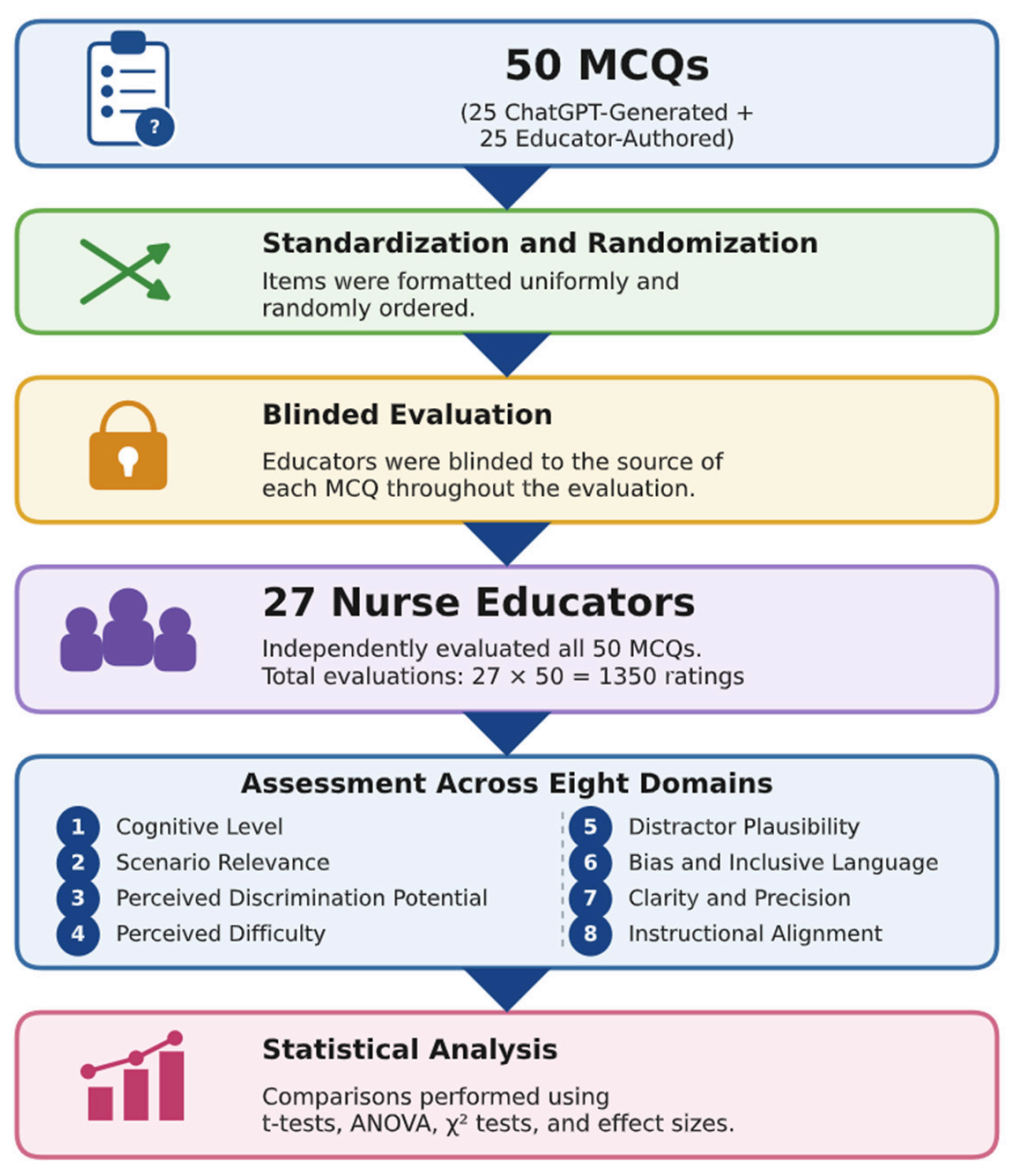
Study Workflow and Blinded Evaluation Process.

Figure 1 provides an overview of the study design, blinded evaluation process, and analytic framework. All analyses were conducted using SPSS version 27 (IBM Corp., Armonk, NY, USA).

## 3. Results

### 3.1. Participant Characteristics

As shown in Table 1, twenty-seven nurse educators participated in the study. Nearly half (48.1%) were aged 45–55 years, two-thirds (66.7%) held doctoral qualifications, one-third (33.3%) reported more than 21 years of teaching experience, and 74.1% had previous experience using ChatGPT.

### 3.2. Comparison Between ChatGPT-Generated and Educator-Authored MCQs

**Figure 2:**
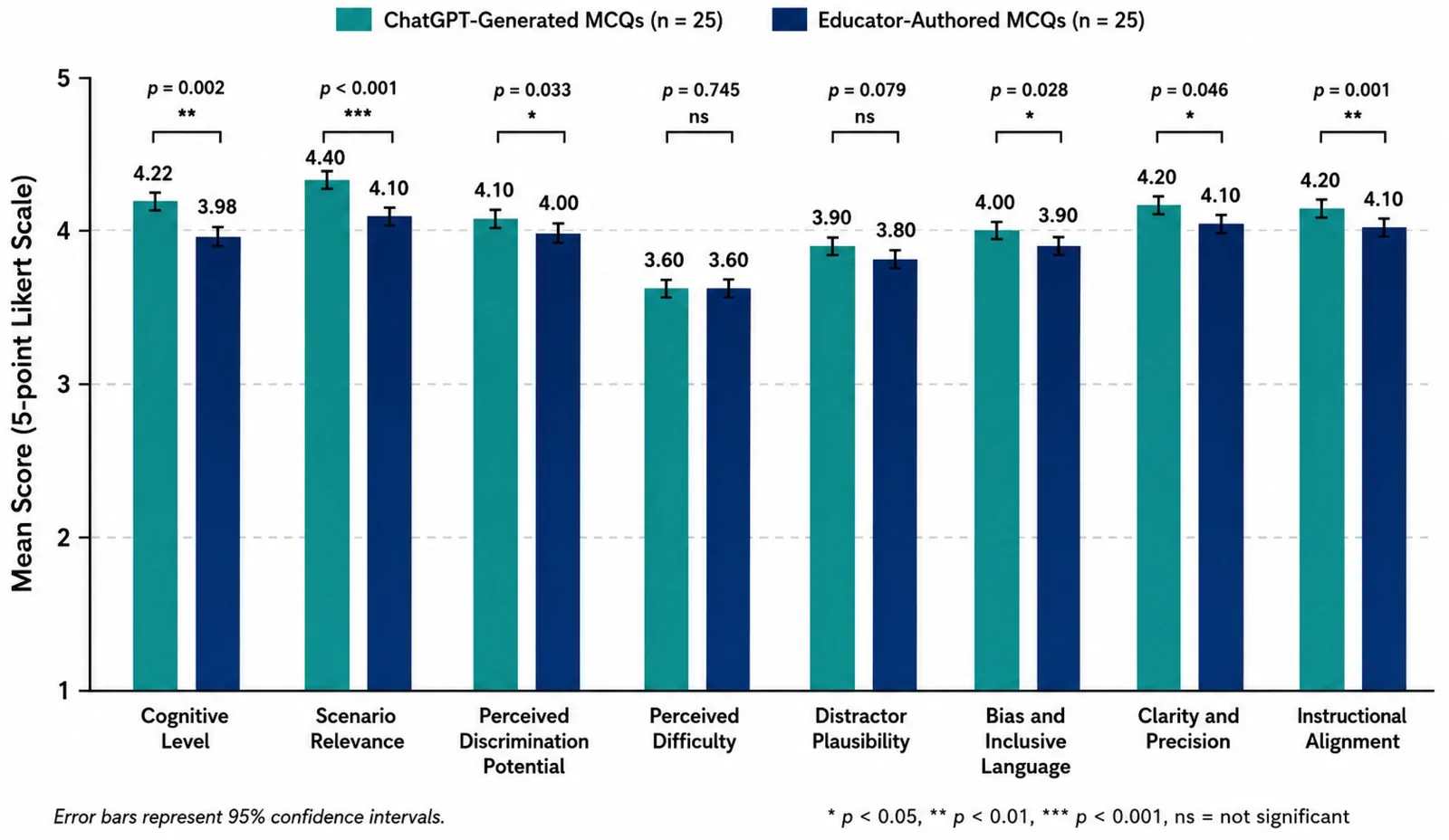
Mean Quality Ratings of ChatGPT-Generated and Educator-Authored MCQs Across Eight Assessment Domains.

As shown in Table 2, ChatGPT-generated items received generally high ratings, with mean domain scores ranging from 3.6 to 4.4. ChatGPT-generated questions achieved nominally higher ratings than educator-authored items in cognitive level, scenario relevance, perceived discrimination potential, bias and inclusive language, clarity, instructional alignment, and overall quality score; after Bonferroni correction for the eight domain comparisons, only the differences in cognitive level, scenario relevance, and instructional alignment held up, while those in perceived discrimination potential, bias and inclusive language, and clarity and precision did not survive correction. All effect sizes were small (Cohen’s *d* = 0.11–0.33), with the largest difference observed for scenario relevance (d = 0.33), indicating substantial overlap between the two item sources. No significant differences were observed in perceived difficulty or distractor plausibility. Figure 2 displays the mean quality ratings for ChatGPT-generated and educator-authored MCQs across the eight assessment domains. Fewer than 3.5% of the 1350 item-level ratings were missing (*n* < 47); consistent with the procedure described in the Methods, these missing values were imputed using the item-level mean for the corresponding domain. The values reported in Table 2 are therefore based on the imputed dataset.

### 3.3. Association Between Age and MCQ Quality Ratings

Table 3 shows significant differences in overall MCQ quality ratings were observed across age groups. Educators aged 35–45 years assigned the highest overall quality ratings (33.8 ± 5.0), followed by those aged 45–55 years (32.7 ± 6.1). Lower ratings were reported by educators in the youngest age group (30.3 ± 5.0) and the oldest age group (29.0 ± 4.8). One-way ANOVA revealed a statistically significant association between age group and overall quality ratings (F = 44.66, *p* < 0.001, η^2^ = 0.091), indicating that age has a moderate effect on the ratings given to the MCQs.

### 3.4. Association Between Teaching Experience and MCQ Quality Ratings

There was no statistically significant impact of the teaching experience of the participants on the ratings given to the MCQs. The participants with 11 to 20 years of teaching experience gave the highest mean rating of 32.6 (standard deviation [SD] = 6.2) to the quality of the MCQs. Participants with more than 21 years of experience gave the second-highest mean rating of 32.0 (SD = 6.1). Participants with 6 to 10 years and 0 to 5 years of teaching experience gave similar ratings of 31.8 (SD = 3.5) and 31.7 (SD = 5.9), respectively. The one-way ANOVA for teaching experience showed an F-value of 1.69 and a *p*-value of 0.167. The eta-squared value of 0.004 indicated a very small effect of teaching experience on the quality ratings given to the MCQs. There was no statistically significant difference in the mean quality ratings given to the MCQs according to the participants’ teaching experience (Table 4).

### 3.5. Perceived Suitability of MCQs for Nursing Examinations

Overall, 87.2% of item evaluations classified the MCQs as suitable for inclusion in nursing examinations, whereas 12.8% were considered unsuitable (Table 5). This finding suggests broad acceptance of both ChatGPT-generated and educator-authored items among the expert evaluators. To better understand the basis of these judgments, domain-specific comparisons between suitable and unsuitable items are provided in Section S4 (Supplementary Materials).

### 3.6. Accuracy of Identifying MCQ Source

Participants could correctly identify the source of MCQs 51.4% of the time (Table 6a). In other words, participants performed no better than chance at identifying whether the MCQ had been created by humans or by ChatGPT. In addition, participants frequently misclassified the source of the MCQs. Table 6b demonstrates that the two types of questions were not perceived to have different characteristics. A one-sample proportion test comparing the observed 51.4% accuracy against the chance value of 50% was not statistically significant (χ^2^(1) = 1.02, *p* = 0.31), confirming that assessors performed no better than chance. Because this pooled test treats the 1350 classifications as independent, it is reported as a supplementary, exploratory check rather than a confirmatory analysis.

**Figure 3:**
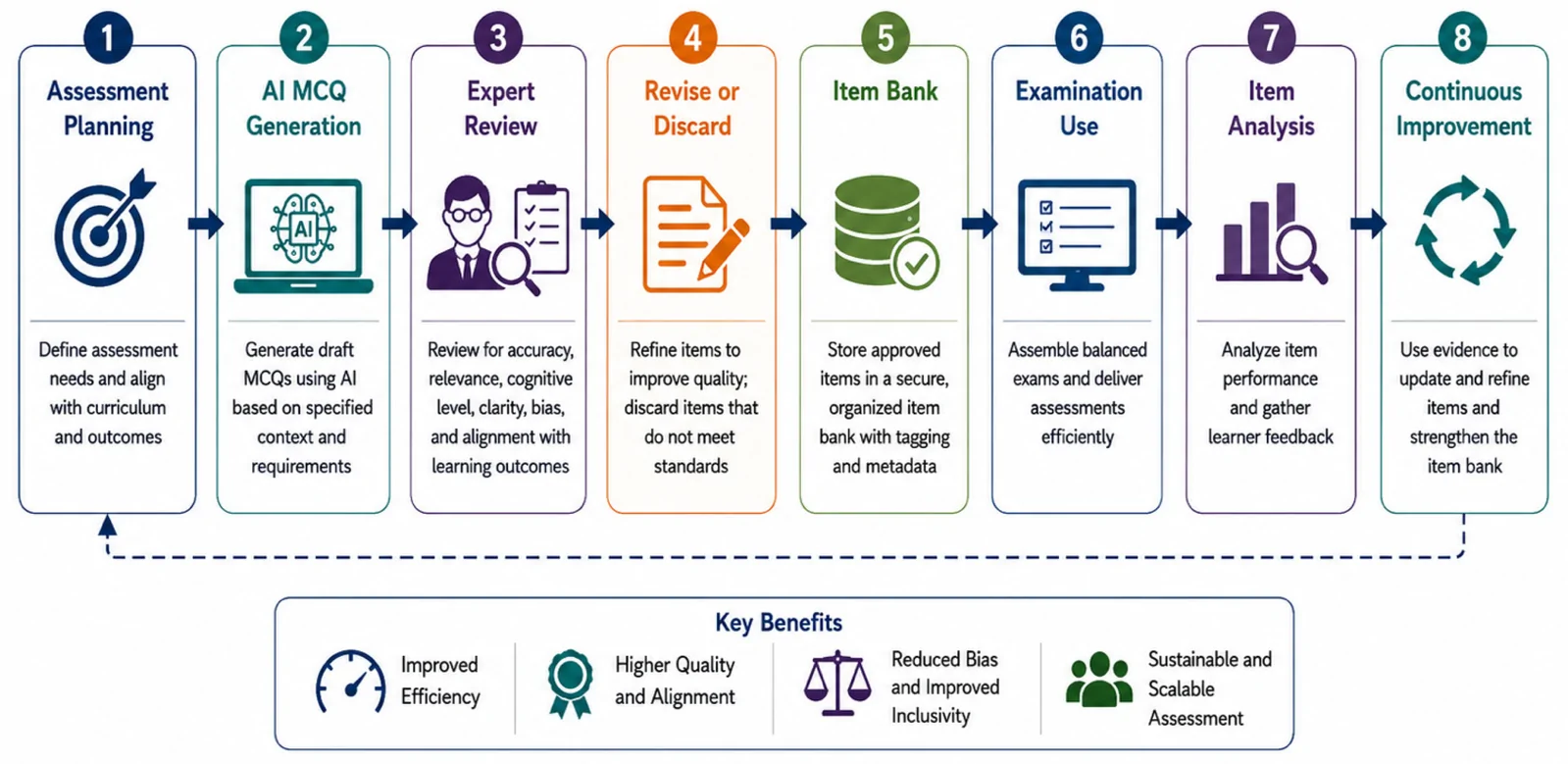
Proposed educator–AI collaborative model for MCQ development and quality assurance. This is a conceptual model derived from the study findings, not an empirical result. The solid arrows indicate the progression through the stages and the dashed arrow indicates the iterative feedback from Continuous Improvement to Assessment Planning.

Based on these findings, a conceptual educator–AI collaborative model is proposed and presented as Figure 3 in the Discussion (Section 4.5); it should be read as a proposed model rather than an empirical result.

## 4. Discussion

The present findings challenge a common assumption in nursing education that AI-generated assessment items are primarily suitable for factual recall or summarization [14]. Contrary to expectations that AI-generated questions may be limited to factual recall, ChatGPT-generated MCQs achieved slightly higher ratings for cognitive level and scenario relevance, suggesting that carefully designed prompts can support the generation of assessment items perceived as targeting higher-order thinking [8,16].

Importantly, these findings do not indicate that AI independently produces superior assessment content. Rather, they reflect the performance of AI-generated items after systematic refinement and quality screening. Consequently, the results support the potential value of educator-supervised, AI-assisted item development rather than fully automated assessment generation [9,22].

### 4.1. Can Experts Distinguish AI-Generated from Human-Authored Items?

A striking finding of this study was that nurse educators, typically expected to have the skills to assess the quality of exam questions, were only able to accurately identify the author of the questions 51.4% of the time. This accuracy did not differ significantly from the chance level of 50% (or flipping a coin?). The AI-generated questions were often classified as being written by the educator, while the questions written by educators were often classified as being AI-created. This suggests that expert educators were not able to identify the source of the questions with any accuracy beyond the level of chance. This finding is notable considering that these educators were experienced individuals with a background in the topic. The finding suggests that AI-created questions are becoming increasingly similar to questions created by human authors. This suggests the potential for challenges with AI-generated exam questions and their governance in the future. The findings of this study have two significant implications. First, the fact that the experts were unable to differentiate between AI-created and educator-created questions indicates that the use of a blinded procedure in this study successfully ensured the validity of the quality assessment of the questions. Second, given that experts were unable to distinguish the authors of the questions, it suggests the potential benefits of utilizing AI to assist with the development of exam questions while recognizing the challenges associated with the use of AI in education and the need to establish policies related to AI-assisted exams to maintain the academic integrity of those exams.

### 4.2. Why Did AI Score Higher?

One plausible reason for the higher ratings for AI-generated MCQs is the use of a standardized structure that large language models provide. Unlike human item writers, AI language models like ChatGPT follow the instructions given to generate their items [16,23]. Iterating through the prompts given to the AI language model may also have contributed to higher ratings from the AI-generated MCQs [15]. Overall, there is a significant benefit to using structured prompts to guide the creation of any type of item from an AI language model [24,25,26,27,28].

Another reason for the higher ratings for the AI-generated MCQs may be due to the fact that they are more consistent than human-written MCQs. While human writers may use different styles of writing and different levels of expertise to construct each MCQ, language models follow the instructions and constraints given to them consistently. The higher consistency of AI-generated items may have led to the higher ratings for those questions in those specific categories [2,23].

A further possible reason for the higher ratings given to the AI-generated MCQs is that they are more aligned with the curriculum outcomes and cognitive levels that were given to the AI language model to generate those questions in the first place. Human educators may have their own understanding of the curriculum outcomes and cognitive levels of the students, but by specifying these outcomes and cognitive levels in the prompt given to the AI language model, the resulting items are more likely to align with the rubric in terms of instructional alignment and cognitive levels [8,10]. The effect of this may be more apparent in the rating categories where the AI language model was expected to score higher due to the structured nature of the items and their prompting process. A simpler explanation may be the difference in review intensity between the two sources: ChatGPT-generated items underwent iterative prompting and expert refinement, while each educator-authored item received a single review pass. Under this explanation, the observed differences may reflect how many rounds of refinement an item received more than any difference attributable to the source itself.

### 4.3. Comparison with the Literature

The present results are broadly consistent with the growing body of literature indicating that, when used correctly, AI-generated MCQs can achieve perceived quality comparable to, and in some studies rated more favorably than, human-generated questions [25,28]. Furthermore, in the field of health professions, the quality of ChatGPT-generated questions is frequently reported to be comparable to human-authored questions [16,25,28], though direct comparison across studies is complicated by differences in the specific AI model or version used, the prompting strategy, the discipline and educational level assessed, and the evaluation method (blinded expert rating versus psychometric item analysis).

However, the results of these studies have been inconsistent. Other studies have indicated that human-written questions are either as good as, or better than, AI-generated questions [11,12]. Additionally, one study found that even though it took AI a short time to generate questions, they lacked the same level of depth as those that would have been required of human experts creating the questions for the assessment tasks [13]. Similarly, another study indicated that while some AI-generated questions were high in quality, the majority still needed significant human revision before use in educational settings [14,26]. These other differences between these studies may be due to the way the prompts were created for the AI, the disciplines in which the questions were created, the experience of the evaluators, the AI model used, and the degree of human review applied before the question of quality evaluations took place. Overall, the study that resulted in the favorable evaluations of AI-generated MCQs had humans reviewing and re-prompting the AI to ensure the MCQs were of high enough quality for use in an assessment task. These individuals examined for the accuracy of the content of the MCQs, the clarity of the wording used, the relevance of the MCQs to the content of the course in which they were to be used, the level of cognitive skill that the MCQs would require from the students, the plausibility of the distractors created by the AI, the presence of bias or non-inclusive language in the MCQs, and the alignment between the MCQs and the course objectives.

### 4.4. Assessor Characteristics

The results of the study showed some differences between the quality ratings assigned to the MCQs based on the age and teaching experience of the evaluators [18,19,29,30,31]. The findings related to the age and teaching experience of the evaluators should be cautiously interpreted due to the nature of the study.

With regard to the age of the evaluators, it was noted that educators in the age ranges of 35–45 years old and 45–55 years old assigned higher overall quality ratings than their younger and older colleagues [19]. However, the youngest (*n*  =  2) and oldest (*n*  =  5) age groups were small; therefore, these findings should be interpreted cautiously and considered hypothesis-generating rather than evidence of a true age-related effect. Future studies with larger and more balanced age groups are needed to examine this relationship.

Regarding the teaching experience of the evaluators, while some found differences between the quality ratings of the MCQs based on teaching experience in particular areas of assessment, such as the potential for discrimination in MCQs, the perceived difficulty of the MCQs, the potential for bias and the inclusion of inclusive language, and the clarity of the MCQs, there was no finding of differences in overall quality ratings based on teaching experience [29,31]. For instance, even though educators with less teaching experience provided more positive evaluations of the MCQs for clarity and inclusion of non-discriminatory language, those with more experience provided more positive evaluations of the MCQs for their potential for discrimination and difficulty.

While these findings are presented as exploratory, given the size of the various subgroups of educators and the study design, there is some reassurance of the generalizability of the use of expert panels in the assessment of MCQs. It is recommended that further studies be conducted in order to more fully investigate the findings with regard to the impact of the age of the evaluators on the quality ratings assigned to MCQs.

### 4.5. Educational Significance

The significance of these findings cannot be overlooked. As educational programs in nursing continue to face challenges regarding faculty workloads, curriculum expansion and the need to implement competency-based assessments, the need for substantial item banks is ever present. AI technology can be used to reduce the workload associated with generating examination items while still maintaining the desired levels of quality. Thus, instead of replacing the educator, AI can be incorporated into the learning environment as a means of augmenting the ability of educators to focus on tasks that are high-value for them. Such a finding is especially relevant for graduate nursing programs, as these programs often encounter faculty shortages while experiencing a need to prepare students for the next stage of their careers with high-quality examinations. As a result, the findings of this research are significant for the field of nursing education, as it provides evidence that AI-related item generation methods can support the development of examinations with the desired quality while improving the overall efficiency of the educational programs.

Given how small the quality differences between sources were, and that assessors could not reliably distinguish item origin, Figure 3 proposes a conceptual educator–AI collaborative model for MCQ development and quality assurance; this model is offered as a proposed framework for future implementation and evaluation, not as an empirical finding of this study.

Study Contribution to Nursing Education

This study contributes to nursing education by providing one of the first blinded evaluations of AI-generated graduate-level MCQs using a multidimensional expert assessment framework. Beyond comparing perceived item quality, the study found that experienced educators were generally unable to distinguish AI-generated from educator-authored questions under blinded conditions—a finding that speaks both to the evolving capabilities of large language models and to the continuing need for expert human oversight in assessment development.

### 4.6. Strengths and Limitations

Strengths

This study has several methodological strengths. Evaluators were blinded to item source throughout the rating process (Section 2.5), which limited the risk that expectations about AI versus human authorship influenced the ratings. Both item sources were developed against the same graduate nursing course objectives and underwent a common formatting-standardization step before evaluation (Section 2.4). The eight-domain rubric captured several clinically relevant dimensions of item quality rather than a single global score, and the panel included experienced graduate nurse educators from multiple institutions. The Supplementary Materials (Sections S1–S4) also document the item-writing instructions, rubric anchors, and ChatGPT prompting procedure in detail, supporting transparency and reproducibility.

Limitations

Certain limitations were identified within the results of the study. First, the study used an exploratory design within expert panels. The number of educators participating in the study was relatively small. As a result, there is a limitation to the generalizability of the study’s results. Second, there were a limited number of educators and items used within the study. As a result, standard statistical tests may have underestimated the error margins of the study’s results. A multilevel statistical approach would be needed to study the error margins appropriately in future studies. Third, the quality rubric developed for the study was not externally validated. Fourth, all examination items were generated using the GPT-4 AI model. As a result, the study’s results may not be generalizable to other AI-related models used in the creation of examination items. In addition, only one set of items was used to represent the graduate nursing programs. As a result, there is a limitation to the transferability of these results to other programs. Finally, rather than using student performance data to assess the quality of the created items, the quality of those items was assessed according to the opinions of a group of nurse educators. One limitation of the created educator-authored questions is that they were generated according to various nursing texts and course outcomes. The texts and resources used in the creation of the ChatGPT-generated questions were not specified; therefore, the sources of those items cannot be traced. As a result, there may be inherent biases in the examination items. In addition, since the examination items created by educators were generated by more than one educator, they may include items that reflect various perspectives and qualities of the individual educators. Therefore, variability between the educators who produced the examinations may be inherent in the results of the study. A further limitation is the asymmetry in review intensity between the two item sources noted in Section 2.4 and Section 4.2: ChatGPT-generated items underwent iterative prompting and refinement, whereas educator-authored items each received a single review pass; this difference, rather than authorship source per se, may account for some of the observed rating differences. One participant’s highest qualification was a BSN rather than a graduate nursing degree (Section 2.2); although this individual met the study’s other eligibility criteria, this represents a minor deviation from the stated eligibility criterion. Finally, given the nested structure of the rating data described in Section 2.7, all analyses involving assessor age and teaching experience, the Bonferroni-corrected domain comparisons, and the chance-level test of source-identification accuracy should be interpreted as exploratory rather than confirmatory.

### 4.7. Practical Implications for Nursing Education

The findings of this exploratory study suggest a few practical implications for nursing education. Individual educators might use AI tools such as ChatGPT to draft an initial MCQ around a specified topic, cognitive level, and clinical scenario, then review that draft against the eight quality domains used in this study, editing or discarding items that score poorly—particularly on perceived difficulty and distractor plausibility, where neither source showed a meaningful advantage. Universities and nursing programs might use AI-assisted item generation to build shared item banks, ease the time burden of expanding course content, and incorporate effective prompting and mandatory human review into faculty development before items are used in high-stakes examinations [32]. For researchers, priorities include establishing the psychometric performance of AI-generated items using actual student response data, replicating these findings with mixed-effects models that account for the nested rating structure, and comparing multiple large language models and prompting strategies. Across these applications, the common thread is that AI can plausibly reduce the workload of initial item generation, but human expert review remains a necessary step before items are used for high-stakes assessment [22].

### 4.8. Future Research

A few directions for future research follow from these findings and from the limitations described in Section 4.6. Replicating this comparison using crossed mixed-effects models, with random effects for assessors and items, would properly account for the nested rating structure and yield more precise estimates of the between-source differences. It would also be valuable to administer both ChatGPT-generated and educator-authored items to actual students and evaluate them using item response theory—including item difficulty, discrimination, and reliability—to establish whether the perceived-quality advantages observed here translate into genuine psychometric performance. Longitudinal evaluation across multiple examination cycles would help clarify whether these findings hold stable over time and across changing model versions, and comparative studies involving multiple large language models, a range of prompting and refinement strategies, and diverse health-profession disciplines and educational levels would help determine how generalizable these findings are. More broadly, this line of research intersects with questions of AI governance and assessment integrity in nursing education, including how institutions verify the provenance of AI-assisted items, how much human review is sufficient before an item is used in a high-stakes examination, and how programs should disclose AI involvement in assessment development to students and accreditation bodies.

## 5. Conclusions

This study suggests that ChatGPT can support the development of graduate nursing multiple-choice questions with perceived quality comparable to educator-authored items. However, these findings are based on expert evaluation rather than objective psychometric performance and should be interpreted cautiously.

AI may serve as a collaborative tool to support, rather than replace, educator judgment in assessment development. Future studies should evaluate AI-generated items using larger and more diverse samples, student performance data, rigorous psychometric methods, and statistical approaches that account for repeated ratings and item-level variation. Further research across different large language models, health professions, and educational levels is also needed to clarify the appropriate role of AI in assessment development.

## Figures and Tables

**Table 1:** Characteristics of Nurse Educator Participants (*N* = 27).

Characteristic	*n*	%
Age group		
<35 years	2	7.4
35–45 years	7	25.9
45–55 years	13	48.1
>55 years	5	18.5
Teaching experience		
0–5 years	5	18.5
6–10 years	4	14.8
11–20 years	9	33.3
>21 years	9	33.3
Highest qualification		
BSN	1	3.7
MSN	8	29.6
PhD	18	66.7
Prior ChatGPT use		
Yes	20	74.1
No	7	25.9

Note. BSN = Bachelor of Science in Nursing; MSN = Master of Science in Nursing; PhD = Doctor of Philosophy.

**Table 2:** Comparison of Quality Ratings Between ChatGPT-Generated and Educator-Authored MCQs.

Domain	ChatGPT M ± SD	Educator M ± SD	Cohen’s *d*	*p*
Cognitive level	4.22 ± 0.84	3.98 ± 0.98	0.26	<0.001
Scenario relevance	4.40 ± 0.80	4.10 ± 1.00	0.33	<0.001
Perceived discrimination potential	4.10 ± 0.80	4.00 ± 1.00	0.11	0.007
Perceived difficulty	3.60 ± 0.90	3.60 ± 0.90	0.00	0.562
Distractor plausibility	3.90 ± 0.90	3.80 ± 1.00	0.11	0.148
Bias and inclusive language	4.00 ± 0.90	3.90 ± 1.00	0.11	0.022
Clarity and precision	4.20 ± 0.90	4.10 ± 1.00	0.11	0.026
Instructional alignment	4.20 ± 0.90	4.10 ± 1.00	0.11	0.003
Overall quality score	32.7 ± 5.2	31.6 ± 6.2	0.19	<0.001

Note. Ratings on a 5-point Likert scale (1 = very poor to 5 = excellent). Total ratings = 1350 (675 ChatGPT + 675 educator-authored). Overall quality score = sum of the eight domains (possible range 8–40). Cohen’s *d* was calculated from the reported group means and pooled standard deviations and should be regarded as exploratory given the nested data structure. Cohen’s *d* values of 0.20 indicate small effects, 0.50 medium effects, and 0.80 large effects. Abbreviations: M, mean; SD, standard deviation; MCQs, multiple choice questions. 95% CIs for Cohen’s *d*, cognitive level 0.15–0.37; scenario relevance 0.22–0.44; perceived discrimination potential 0.00–0.22; perceived difficulty −0.11–0.11; distractor plausibility 0.00–0.22; bias and inclusive language 0.00–0.22; clarity and precision 0.00–0.22; instructional alignment 0.00–0.22; overall quality score 0.08–0.30.

**Table 3:** MCQ Quality Ratings According to Assessor Age.

Age Group	Educators (*n*, %)	Ratings (*n*, %)	Overall Quality M ± SD
<35 years	2 (7.4%)	100 (7.4%)	30.3 ± 5.0
35–45 years	7 (25.9%)	350 (25.9%)	33.8 ± 5.0
45–55 years	13 (48.1%)	650 (48.1%)	32.7 ± 6.1
>55 years	5 (18.5%)	250 (18.5%)	29.0 ± 4.8
Total	27	1350	32.1 ± 5.8

Note. Ratings (*n*) = pooled item-level evaluations; each of the 27 educators independently rated all 50 MCQs (27 × 50 = 1350). One-way analysis of variance (ANOVA) for the overall quality score: F(3, 1346) = 44.66, *p* < 0.001, η^2^ = 0.091. Full domain-by-domain ANOVA results, including η^2^, are reported in Supplementary Table S1. MCQs, multiple choice questions; SD, standard deviation.

**Table 4:** MCQ Quality Ratings According to Teaching Experience.

Teaching Experience	Educators (*n*)	Ratings (*n*)	Overall Quality M ± SD
0–5 years	5	250	31.7 ± 5.9
6–10 years	4	200	31.8 ± 3.5
11–20 years	9	450	32.6 ± 6.2
>21 years	9	450	32.0 ± 6.1
Total	27	1350	32.1 ± 5.8

Note. Ratings (*n*) = pooled item-level evaluations; each of the 27 educators independently rated all 50 MCQs. One-way analysis of variance (ANOVA) for the overall quality score: F(3, 1346) = 1.69, *p* = 0.167, η^2^ = 0.004, indicating no significant association between teaching experience and overall quality ratings. Full domain-by-domain ANOVA results, including η^2^, are reported in Supplementary Table S2. MCQs, multiple choice questions; SD, standard deviation.

**Table 5:** Perceived Suitability of MCQs for Inclusion in Nursing Examinations.

Suitability Rating	*n*	%
Suitable	1177	87.2
Not suitable	173	12.8
Total	1350	100

Note. Suitability was assessed using the question, “Do you consider this MCQ suitable for inclusion in nursing examinations?” Percentages are based on all 1350 item-level evaluations. MCQs, multiple choice questions.

**Table 6:** (**a**) Accuracy of Identifying the Source of MCQs. (**b**) Cross-Classification of Actual Source and Assessor Identification.

(a)		
Classification Outcome	*n*	%
Correct identification	694	51.4
Incorrect identification	656	48.6
Total	1350	100
(b)		
Actual source	Classified as ChatGPT- generated	Classified as educator- authored
ChatGPT-generated	400 (row 59.3%; col 51.2%)	275 (row 40.7%; col 48.3%)
Educator-authored	381 (row 56.4%; col 48.8%)	294 (row 43.6%; col 51.7%)

Note. Correct identification was defined as agreement between the actual source of the MCQ and the participant’s classification. MCQs, multiple choice questions. Note. Participants were blinded to item source throughout the evaluation process. Values represent the frequency of classifications across all 1350 evaluations. The diagonal cells (400 + 294 = 694) represent correct classifications and correspond to the overall accuracy reported in Table 6a. Row percentages are calculated within each actual-source row (*n* = 675 per row); column percentages are calculated within each identification column (Identified as ChatGPT, *n* = 781; Identified as educator, *n* = 569).

## Data Availability

The data that support the findings of this study are available from the corresponding author upon reasonable request.

## Supplementary Materials

The following supporting information can be downloaded at https://www.mdpi.com/article/10.3390/nursrep16090304/s1, Supplementary Table S1: Domain-by-Domain One-Way ANOVA of MCQ Quality Ratings by Assessor Age; Supplementary Table S2: Domain-by-Domain One-Way ANOVA of MCQ Quality Ratings by Teaching Experience; Section S1: Instructions for Human Question Writers; Section S2: Rubric Domains and Rating Anchors; Section S3: ChatGPT Prompt Templates and Examples; Section S4: Qualitative Examples of Unusable ChatGPT-Generated Items.

## Public Involvement Statement

No public involvement in any aspect of this research.

## Guidelines and Standards Statement

This manuscript was drafted against the STROBE (Strengthening the Reporting of Observational Studies in Epidemiology) statement for cross-sectional studies.
