# Supplementary material for: Can AI Write MCQs? A Comparison of ChatGPT-Generated and Educator-Authored Multiple-Choice Questions in Graduate Nursing Education
Source: Nurs Rep. 2026 Aug 27;16(9):304. doi: 10.3390/nursrep16090304 (PMC13610188; doi:10.3390/nursrep16090304)
Supplement: Supplementary file 1 [file nursrep-16-00304-s001.zip › nursrep-4469293-supplementary.pdf]

Supplementary Material

Supplementary Table S1. Domain-by-Domain One-Way ANOVA of MCQ Quality Ratings by Assessor Age.

Quality domain	F	p	η^2
Cognitive level	17.75	< .001	0.038
Scenario relevance	26.16	< .001	0.055
Perceived discrimination potential	17.52	< .001	0.038
Perceived difficulty	0.57	.635	0.001
Distractor plausibility	44.22	< .001	0.090
Bias and inclusive language	55.93	< .001	0.111
Clarity and precision	65.62	< .001	0.128
Instructional alignment	47.55	< .001	0.096
**Overall quality score**	**44.66**	**< .001**	**0.091**

Note. One-way ANOVA across the four age groups (df = 3, 1346) for each quality domain and for the overall quality score. η^2 (eta squared) is reported as an effect-size estimate ($\eta^2 \approx 0.01$ small, 0.06 medium, 0.14 large). Values were computed from the group means, SDs, and cell sizes and should be confirmed with mixed-effects models given the nested data structure.

Supplementary Table S2. Domain-by-Domain One-Way ANOVA of MCQ Quality Ratings by Teaching Experience.

Quality domain	F	p	η^2
Cognitive level	1.79	.147	0.004
Scenario relevance	1.06	.364	0.002
Perceived discrimination potential	5.07	.002	0.011
Perceived difficulty	17.86	< .001	0.038
Distractor plausibility	1.06	.367	0.002
Bias and inclusive language	9.09	< .001	0.020
Clarity and precision	6.44	< .001	0.014
Instructional alignment	1.85	.137	0.004
**Overall quality score**	**1.69**	**.167**	**0.004**

Note. One-way ANOVA across the four teaching-experience groups (df = 3, 1346) for each quality domain and for the overall quality score. η^2 is reported as an effect-size estimate ($\eta^2 \approx 0.01$ small, 0.06 medium, 0.14 large).

Sections (Supplementary Material)

The following materials are provided as online supplementary material: the standardized instructions issued to human item writers (Section S1), the rubric domain definitions and rating anchors (Section S2), the ChatGPT prompt templates used for AI generation (Section S3), and illustrative examples of items judged unusable (Section S4), together with Supplementary Tables S1 and S2.

Section S1. Instructions for Human Question Writers.

Title: Standardized Instructions for Human-Authored MCQs

You are invited to develop multiple-choice questions (MCQs) for graduate-level nursing examinations. Please follow the instructions below to ensure consistency with AI-generated items and alignment with the study objectives.

1. Target learner level

Target audience: students in a graduate-level nursing program (e.g., MSN, post-graduate diploma). Assume candidates have completed core courses in advanced pathophysiology, pharmacology, and health assessment.

2. Source material

Base your questions on the official course learning outcomes for graduate-level nursing courses NURS 411 Psychiatric mental health, NURS 413 Legal and ethical issues, NURS 414 Leadership and management, NURS 427 Community Health, NURS 415 Health Education and promotion. Use sources including: Applied Pathophysiology for the Advanced Practice Nurse, 2nd ed.; Legal and ethical issues in nursing, 7th ed.; Effective Leadership and Management in Nursing, 9th ed.; Community and Public Health Nursing: Promoting the Public's Health, 10th ed.; Health Promotion in Nursing Practice, 7th ed. Avoid using or paraphrasing any copyrighted examination items (e.g., NCLEX or other standardized tests).

3. Content and topic selection

Select topics that are core to safe practice in graduate nursing (e.g., management of heart failure, sepsis, diabetes with complications, advanced pharmacotherapeutics, complex patient assessment) and reflect current, evidence-based practice. Distribute items across the main content domains provided in the blueprint so that no single area predominates.

4. Bloom's taxonomy and cognitive level

Each MCQ should be tagged to a Bloom's level at the time of writing. Aim for up to 25% at lower levels (remember/understand), at least 50% at application or analysis, and remaining items at evaluation or higher-level clinical reasoning.

5. Question format

For each MCQ, provide a clear, focused problem or scenario relevant to graduate-level nursing practice. Include exactly four options (A–D), only one of which is clearly correct. Distractors must be plausible and reflect common errors, misconceptions, or alternative-but-inferior clinical choices. Use clear, precise, and bias-free language.

6. Prohibited practices

Do not reuse any existing exam questions, including institutional banks or licensed exams (e.g., NCLEX, specialty certifying exams). Do not include "all of the above" or "none of the above" as answer options.

7. Documentation

For each item, provide the full stem and four options with the correct answer indicated, the intended Bloom's level, the primary content area, and a brief justification (1–2 sentences) explaining why the correct option is preferred over the distractors.

Section S2. Rubric Domains and Rating Anchors.

For each domain (e.g., cognitive level, scenario relevance, discrimination, difficulty, distractors, bias and inclusivity, clarity, instructional alignment), provide a one-sentence definition and a 1–5 anchor description (1 = very poor; 5 = excellent).

Cognitive level

1: Pure factual recall below expected level of graduate students.

3: Mix recall and basic application; partially meets expected cognitive level.

5: Clearly requires application, analysis, or evaluation within a realistic clinical context, fully appropriate for graduate-level assessment.

Repeat similarly for each domain, consistent with your current rubric.

Section S3. ChatGPT Prompt Templates and Examples.

Title: Standardized Prompts Used for AI Question Generation

The following templates and examples were used with ChatGPT (GPT-4) to generate MCQs for this study.

1. Initial knowledge-level prompt template

"You are an expert nursing educator. Using your nursing knowledge and the reference information I provide, write one multiple-choice question for graduate-level nursing students with the following characteristics: standard nursing exam format; four answer options (A–D), only one correct; focus on accurate, evidence-based content; include plausible but clearly incorrect distractors; do not provide explanations or rationales. Topic: [insert specific topic from graduate nursing curriculum]. Target Bloom's level: remember/understand."

2. Higher-order, application-level prompt template

"You are an expert nursing educator. Develop one scenario-based MCQ for graduate-level nursing students with these characteristics: patient-based clinical scenario relevant to advanced practice nursing; requires application of nursing principles to clinical data or a change in patient status; four options (A–D), only one correct, with plausible distractors; focus on decision making or prioritization rather than simple recall. Topic: [insert topic]. Target Bloom's level: apply/analyze."

3. Higher-order, synthesis/clinical reasoning prompt template

"You are an expert nursing educator. Create one graduate-level MCQ that assesses critical thinking and clinical judgment in a complex patient with multiple co-morbidities. The question should include a brief but realistic clinical vignette; require synthesis of information from different physiological systems or medications; require the student to select the most appropriate nursing action, interpretation, or priority; include four options (A–D) with only one correct answer and plausible distractors. Topic: [insert topic]. Target Bloom's level: analyze/evaluate."

4. Example of an iterative refinement sequence (structure only)

Step 1: Use the initial prompt at remember/understand level for topic X.

Step 2: If the output is purely factual recall, re-prompt with: "Revise this question so that it includes a clinical scenario and requires the student to apply the concept to a patient situation rather than recalling a definition."

Step 3: If still low-level, re-prompt with: "Modify the question so that the correct answer requires comparing two possible interventions and selecting the safest or most appropriate option for the specific patient described."

Only items that met the inclusion criteria (relevance to nursing practice, clarity, plausible distractors, appropriate cognitive level, and alignment with Bloom's taxonomy for nursing education) were retained for the final set.

Section S4. Qualitative Examples of Unusable ChatGPT-Generated Items.

To clarify what constituted "unusable" items (alignment rating ≤ 2), we provide de-identified examples and brief rater rationales.

Example 1

Item description: An MCQ asking for recall of the definition of evidence-based practice without any clinical scenario.

Low-rated domain: Instructional alignment = 2.

Raters' rationale: The item targeted simple recall inconsistent with graduate-level outcomes emphasizing appraisal and application of evidence in complex clinical situations and did not map clearly to the exam blueprint.

Implication: Requires redesign to embed evidence use in a realistic scenario and to target higher-order reasoning.

Example 2

Item description: An MCQ presenting a brief clinical vignette in which multiple answer options could plausibly be correct because key diagnostic details were missing.

Low-rated domains: Clarity and precision of language = 2; cognitive level = 2.

Raters' rationale: The ambiguous stem did not clearly point to a single best answer, promoting guessing rather than meaningful discrimination of learner performance.

Implication: Requires clarification of the vignette with specific clinical findings and a more focused question to ensure a single best response.
